# Gene Expression Profiles Accurately Predict Outcome Following Liver Resection in Patients with Metastatic Colorectal Cancer

**DOI:** 10.1371/journal.pone.0081680

**Published:** 2013-12-10

**Authors:** Hiromichi Ito, Qianxing Mo, Li-Xuan Qin, Agnes Viale, Shishir K. Maithel, Ajay V. Maker, Jinru Shia, Peter Kingham, Peter Allen, Ronald P. DeMatteo, Yuman Fong, William R. Jarnagin, Michael D’Angelica

**Affiliations:** 1 Department of Surgery, Memorial Sloan-Kettering Cancer Center, New York, New York, United States of America; 2 Department of Epidemiology and Biostatistics, Memorial Sloan-Kettering Cancer Center, New York, New York, United States of America; 3 Genomic Core Laboratory, Memorial Sloan-Kettering Cancer Center, New York, New York, United States of America; 4 Department of Pathology Memorial Sloan-Kettering Cancer Center, New York, New York, United States of America; 5 Department of Surgery, Michigan State University, East Lansing, Michigan, United States of America; 6 Department of Medicine, Dan L. Duncan Cancer Center, Baylor College of Medicine, Houston, Texas, United States of America; Institute of Medical Science, University of Tokyo, Japan

## Abstract

**Purpose:**

The aim of this study was to build a molecular prognostic model based on gene signatures for patients with completely resected hepatic metastases from colorectal cancer (MCRC).

**Methods:**

Using the Illumina HumanHT-12 gene chip, RNA samples from the liver metastases of 96 patients who underwent R0 liver resection were analyzed. Patients were randomly assigned to a training (n = 60) and test (n = 36) set. The genes associated with disease-specific survival (DSS) and liver-recurrence-free survival (LRFS) were identified by Cox-regression and selected to construct a molecular risk score (MRS) using the supervised principle component method on the training set. The MRS was then evaluated in the independent test set.

**Results:**

Nineteen and 115 genes were selected to construct the MRS for DSS and LRFS, respectively. Each MRS was validated in the test set; 3-year DSS/LRFS rates were 42/32% and 79/80% for patients with high and low MRS, respectively (*p* = 0.007 for DSS and p = 0.046 for LRFS). In a multivariate model controlling for a previously validated clinical risk score (CRS), the MRS remained a significant predictor of DSS (*p* = 0.001) and LRFS (*p* = 0.03). When CRS and MRS were combined, the patients were discriminated better with 3-year DSS/LRFS rates of 90/89% in the low risk group (both risk scores low) vs 42/26% in the high risk group (both risk scores high), respectively (*p* = 0.002/0.004 for DSS/LRFS).

**Conclusion:**

MRS based on gene expression profiling has high prognostic value and is independent of CRS. This finding provides a potential strategy for better risk-stratification of patients with liver MCRC.

## Introduction

Over the last two decades, the management of metastatic colorectal cancer (MCRC) confined to the liver has dramatically changed. Improvements in systemic and regional chemotherapy as well as surgical technology has allowed an increasing number of patients to be candidates for resection with curative intent. [1] Liver resection has been accepted as the most effective therapy for resectable MCRC isolated to the liver with documented 5-year survival rates of up to 58% for well selected patients. [2]–[4] However, even with complete resection, the majority of patients develop recurrence, commonly within the liver remnant, and a high proportion of this group dies within 2–3 years. [5], [6] Therefore, accurate risk stratification is essential to optimize individual decision making in selection of surgery, and use of adjuvant therapies.

Multiple prognostic scoring systems have been proposed using clinical criteria to stratify the risk of recurrence and cancer-related death after liver resection for patients with MCRC. [3], [7]–[10] However most systems are based on single-institutional data and are therefore steeped with bias related to local referral patterns, patient selection and treatment approaches. Indeed, clinical risk scoring systems have not consistently predicted outcomes when tested across institutions [3], and have not generally predicted outcome well enough to effect clinical practice.

Gene microarray technology allows comprehensive analysis of gene expression profiles of tumors, and such “gene signatures” have the potential to be more accurate and consistent biological markers for the prediction of outcomes. Studies have demonstrated that specific gene signatures can be powerful markers to predict long-term outcomes after treatment for various cancer including breast [11]–[13], lung [14], [15], colon [12], [16], [17] and liver. [18] To date, there are no data available regarding gene expression profiles in patients with resected MCRC to the liver.

The aim of this study was to assess gene expression profiles in resected MCRC isolated to the liver in order to develop a more powerful and accurate prognostic biomarker reflecting tumor biology to predict long-term outcomes.

## Methods

### Patient Samples and RNA Extraction

This study was approved by the Memorial Sloan-Kettering Cancer Center (MSKCC) Institutional Review Board (IRB). All the patients provided written consents for tissue procurement and use of them for research purpose. Fresh-frozen tumor specimens of MCRC in resected liver specimens were obtained from our departmental tissue bank on a separate IRB approved tissue harvesting protocol. All archived metastatic tumors in the tissue bank were collected at the time of surgery from patients who underwent liver resection at our institution. The clinical information for corresponding patients including demographics, pathologic findings and stage, perioperative therapy and follow-up data was obtained from a prospectively maintained hepatic resection database and supplemented by medical record review.

The authors’ approach to hepatic resection for metastatic colorectal cancer has been described previously. [2], [6], [8] In general, patients were submitted to operation when a therapeutic benefit was considered likely and treatment of all disease was technically feasible. Preoperative imaging included contrast-enhanced computed tomography of the chest, abdomen and pelvis with additional imaging performed at the discretion of the treating physician. Colonoscopy was performed within 1 year of liver resection. Decisions regarding the use of hepatic arterial infusion (HAI), adjuvant and/or neo-adjuvant systemic chemotherapy were made on an individual basis in conjunction with consulting medical oncologists. [19] Patients were followed with periodic clinical evaluations, serum CEA levels and CT scans of chest/abdomen/pelvis.

Our unit has previously published a clinical risk score (CRS) using preoperative variables in an effort to stratify patients in terms of survival following hepatic resection for MCRC. [8] The factors that comprise the CRS include 1) lymph node status of the primary tumor, 2) disease free interval (<12 months or ≥12 months), 3) serum CEA level prior to liver resection (>200 ng/ml or ≤200 ng/ml), 4) number of hepatic tumors (1 or >1) and 5) tumor size (≤5 cm or >5 cm). Patients receive 1 point for each adverse factor, and the CRS represents the sum. Patients were then divided into two groups: high risk (CRS≥3) and low risk (CRS<3).

The study endpoints were disease-specific survival (DSS), overall recurrence-free survival (RFS) and liver recurrence-free survival (LRFS). DSS was measured from the time of liver resection to death or last follow-up. RFS and LRFS were measured from the time of liver resection to the time when 1^st^ recurrence and liver recurrence was detected, respectively.

From January 2000 through August 2007, a total of 446 specimens from patients who underwent liver resection for MCRC were banked. Patients with extrahepatic metastasis, those who underwent a margin positive resection, those with missing CRS score data and those without adequate follow-up were excluded. After these exclusions, frozen sections of 307 OCT-embedded samples were evaluated. After histological verification under hematoxylin and eosin staining, 187 of them were found to have at least 70% viable tumor cells. Those frozen tissue samples were macrodissected from the selected area of OCT block and RNA was extracted using TRIzol reagent (Invitrogen, Carlsbad, CA) as recommended by the manufacturer. The RNA quality of these 187 samples was then assayed with a 2100 Bioanalyzer (Agilent Technology, Palo Alto, CA), and 96 of them were found to be optimal quality for microarray analysis with RNA integrity number (RNI) ≥7.

### Microarray Analysis

Extracted total RNA was reverse-transcribed by a previously published method [20] and the resulting complimentary DNA template was applied to gene expression analysis. The target cDNAs were hybridized to Illumina HumanHT-12 Gene Chip containing a total of 47,231 annotated gene probe sets (Illumina, San Diego, CA). Arrays were scanned by using standard Ilumina protocols and scanners. Microarray data are available in the ArrayExpress database (www.ebi.ac.uk/arrayexpress) under accession number E-MTAB-1951. The expression raw data were analyzed using R statistical software, which is available on the Internet at http://cran.r-project.org/.

### Statistical Methods

Patients were randomly assigned to the training and testing sets in a 2∶1 ratio. The data from the training set cohort was used to select prognostic genes and to construct molecular risk scores (MRS), and the data from the testing set cohort was used for its validation.

The microarray data were log2 transformed and quantile normalized. [21] The supervised principal components method was used to identify prognostic gene signatures as described previously. [22] In brief, first, the univariate regression coefficients for each gene associated with specific outcomes (DSS, RFS, or LRFS) in the training data were calculated using the Cox proportional hazards model [23]. Second, the genes significantly associated with the outcome (*p*<0.001) were ranked in the order of their regression coefficient and the top genes with the coefficient about the threshold value were selected as “principle component”. The threshold value was estimated and determined using cross-validation. Third, the first principal component was used to create the MRS, which was defined as the linear combination of weighted expression signals with the standardized Cox’s regression coefficient as the weight.

The patients in the training set cohort were classified into a high and low-risk group based on the median value of the MRS. The median MRS was chosen as the threshold in order to eliminate the effect of extreme values in the training set and to have equal numbers of patients in the high and low risk groups. Survival probabilities were estimated using the method of Kaplan and Meier [24] and compared using the Log-Rank test. Of note, although we attempted to construct an MRS for DSS, RFS and LRFS, we could not calculate an adequately prognostic MRS for RFS. Therefore, the presented analysis focused on DSS and LRFS.

As some patients with MCRC died of extra-hepatic recurrence, the event of liver recurrence and cancer-specific death can compete with each other. To evaluate the effect of cancer death without liver recurrence on gene analysis for liver-specific recurrence, genes associated with liver-recurrence were assessed by two analytical methods; competing risk analysis in which cancer-specific death was considered as competing risk and Cox-regression analysis in which it was considered as censoring. [25].

**Figure 1:**
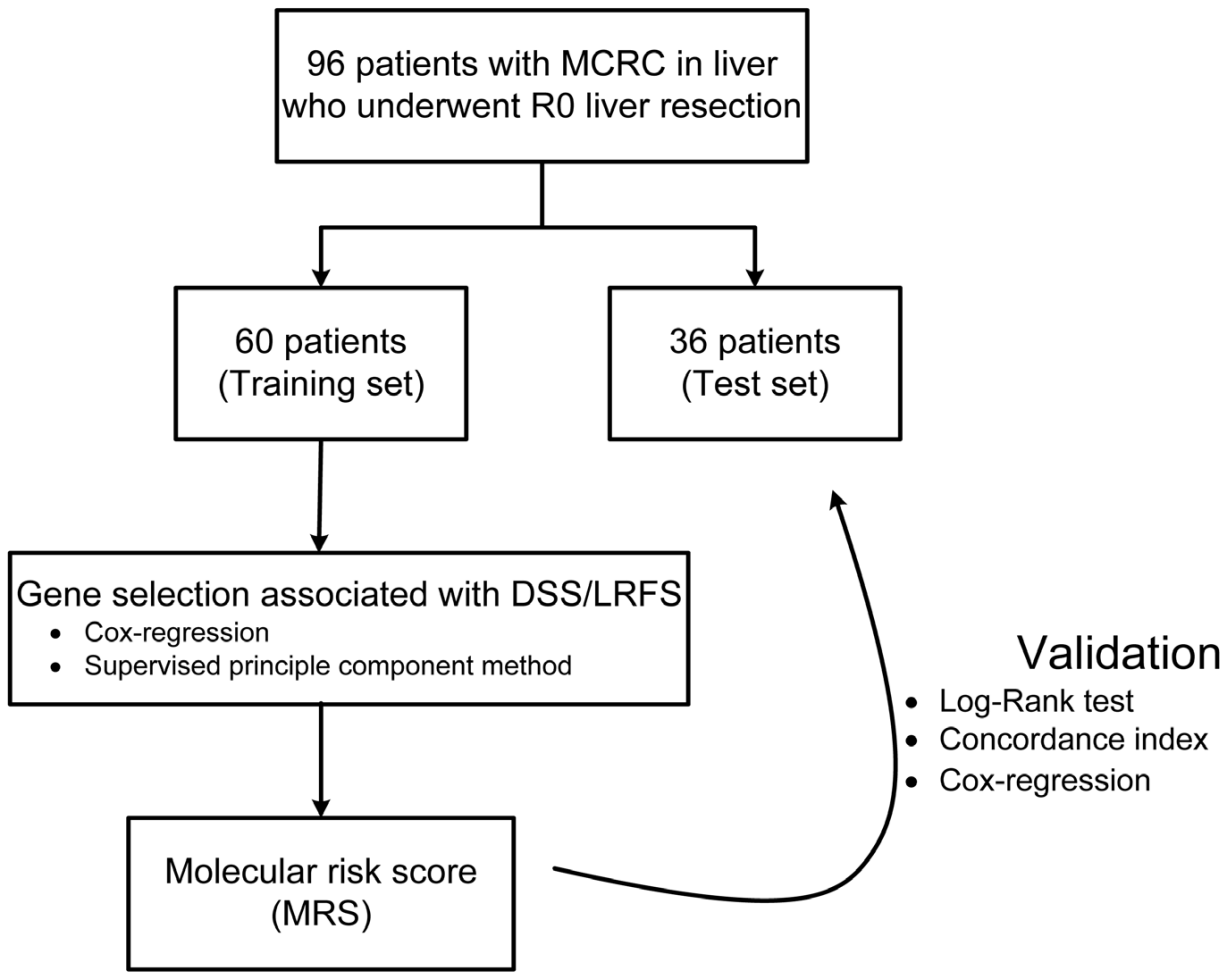
Study profile. Ninety-six samples were selected from our departmental tissue bank. The patients were randomly assigned to the training set and test set by 2∶1. The molecular score was constructed based on the data in the training set cohort and validated using the data in the test set cohort.

For the validation of the MRS models we assessed the discrimination of DSS and LRFS by the MRS when applied to the test set cohort. The MRS and the threshold value derived from the training set cohort were not re-estimated, but directly applied to the test set. Discrimination was quantified using the concordance-index. [26] Concordance indices range from 0 to 1, with a value of 1 indicating that the model perfectly discriminates between patients with higher and lower risk of death (or recurrence), and a value of 0.5 indicating that the predictive ability of the model is no better than chance alone. The statistical relationship between the MRS and CRS was assessed using multivariate Cox-regression analysis. Figure 1 outlines the experimental flow of the study.

Comparison of categorical variables was performed using Fisher exact test, and continuous variables were presented as mean values ± standard error (SE) unless otherwise indicated, and compared using a two-sample *t*-test. A 2-sided *p* value <0.05 was considered significant.

## Results

### Patient Demographics and Clinical Risk Score (CRS)

Of the 96 patients included in the study, 37 (39%) had a CRS ≥3. Sixty-nine patients (72%) received chemotherapy prior to liver resection and 79 (82%) received adjuvant chemotherapy (45 systemic chemotherapy alone, 34 combination of systemic and regional chemotherapy using HAI). Median follow-up was 30 months (range, 2–108 months) for all patients and 39 months (range, 2–108 months) for survivors. During the study period, 66 patients (69%) developed any recurrence and 45 (47%) developed liver recurrence. The liver was the 1^st^ site of recurrence for 29 patients while extrahepatic sites were the first site of recurrence in 37 patients. Thirty-nine patients (41%) died of cancer, 33 of whom (85%) died with documented recurrence in the remnant liver. The patient demographics, tumor characteristics, clinical risk score, type of adjuvant chemotherapy and events rate (cancer death, overall recurrence or liver recurrence during the study period) did not differ significantly between the patients in the training and test sets (Table 1).

**Table 1:** Patient demographics, tumor characteristics and perioperative chemotherapy.

	Training set (N = 60)	Test set (N = 36)	Total (N = 96)	*p* **
Age (median, range)	59 years (29–88)	60 years (34–87)	60 years (29–88)	0.61
Male gender	42 (70)	22 (61)	63 (66)	0.24
*Tumor size >5 cm (%)	16 (27)	6 (17)	22 (23)	0.32
*Primary N+ (%)	35 (58)	22 (61)	57 (59)	0.83
*>1 liver tumor (%)	39 (65)	19 (53)	58 (60)	0.28
*DFI <12 mo	32 (53)	19 (53)	51 (53)	1.0
*CEA >200 (%)	5 (8)	3 (8)	8 (8)	1.0
CRS ≥3 (%)	23 (38)	14 (39)	37 (39)	1.0
Chemotherapy prior to surgery	43 (72)	26 (72)	69 (72)	0.95
Chemotherapy after surgery	47 (78)	32 (89)	79 (82)	0.19
HAI chemotherapy	19 (32)	15 (44)	34 (35)	0.38
Median follow-up	30 mo	29 mo	30 mo	0.97
Cancer death (%)	25 (42)	14 (39)	39 (41)	0.83
Cancer death without liver recurrence (%)#	4 (16)	2 (14)	6 (15)	0.66
Overall recurrence (%)	41 (68)	25 (69)	66 (69)	1.0
Liver recurrence (%)	27 (45)	18 (50)	45 (47)	0.68

CRS: Sum of points for each variable marked as *on the table, ≥3 considered as high risk.

** training set vs test set, ^#^Among overall cancer death.

### Selection of Genes Associated with DSS/LRFS and Construction of Molecular Risk Score (MRS)

**Figure 2:**
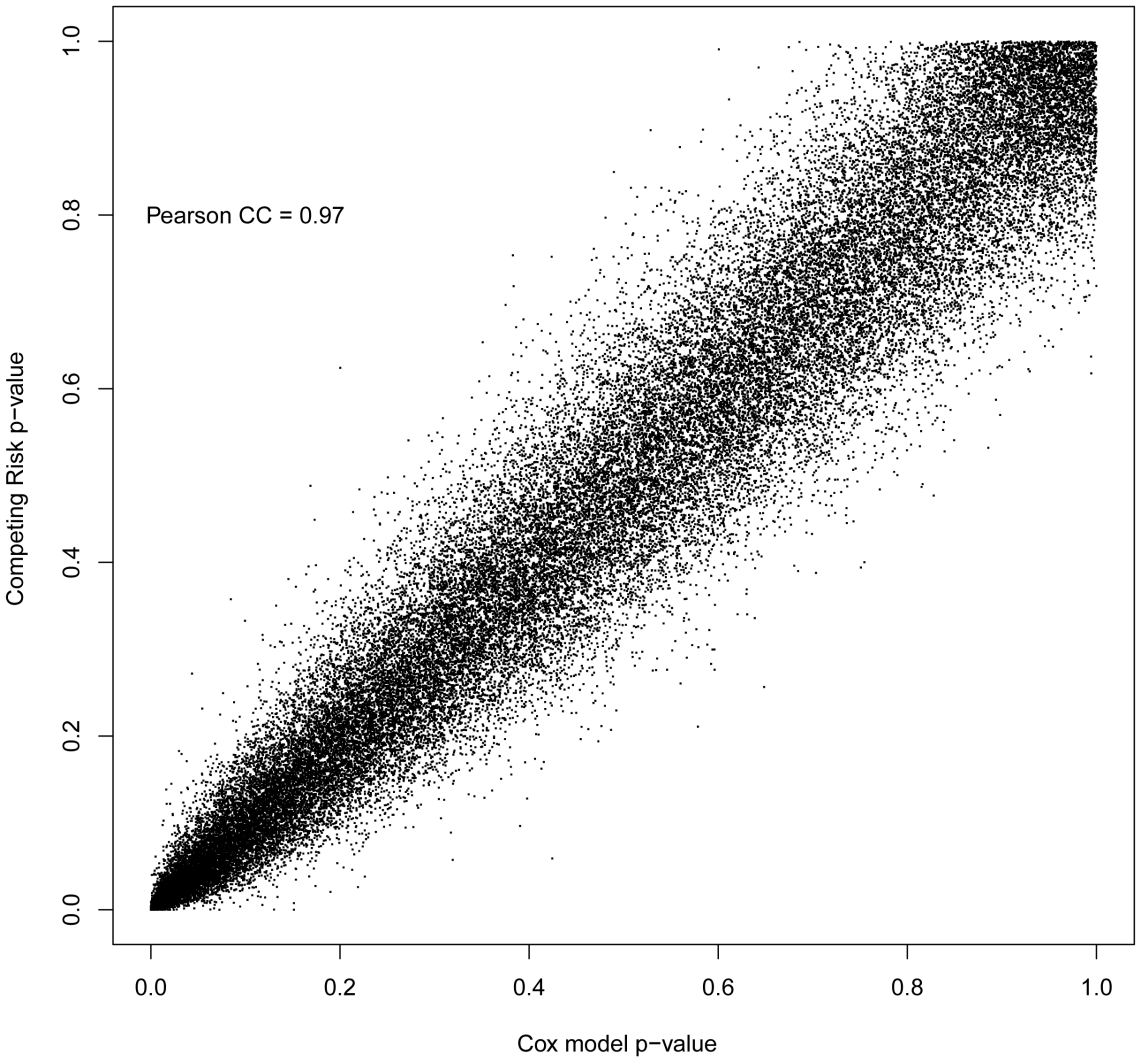
Scatterplot of *p*-values for genes associated with liver-specific recurrence by the competing risk analysis and the Cox-regression analysis. Each dot represents *p*-values for gene in both analyses.

We first analyzed the gene expression data of patients in the training set utilizing the supervised principle component method. Nineteen and 115 gene transcripts were selected as prognostically relevant for DSS and LRFS, respectively. Twelve out of 19 genes (65%) for DSS were also selected among the genes for LRFS. The lists of genes are listed in Table S1. We were unable to select genes and calculate a useful molecular signature for overall recurrence (data not shown). Since liver recurrence and cancer death are potentially competing endpoints, we evaluated the genes associated with liver-specific recurrence using two different analyses; competing risk analysis and Cox-regression analysis. As shown in Figure 2, the two sets of *p*-values for each gene calculated by these two methods were very similar (Peason correlation coefficient [CC] = 0.97). This finding indicated that the competing effect of cancer death without liver recurrence on the gene analysis for LRFS was negligible and therefore we calculated the MRS for LRFS based on the same analysis used to calculate the MRS for DSS.

**Figure 3:**
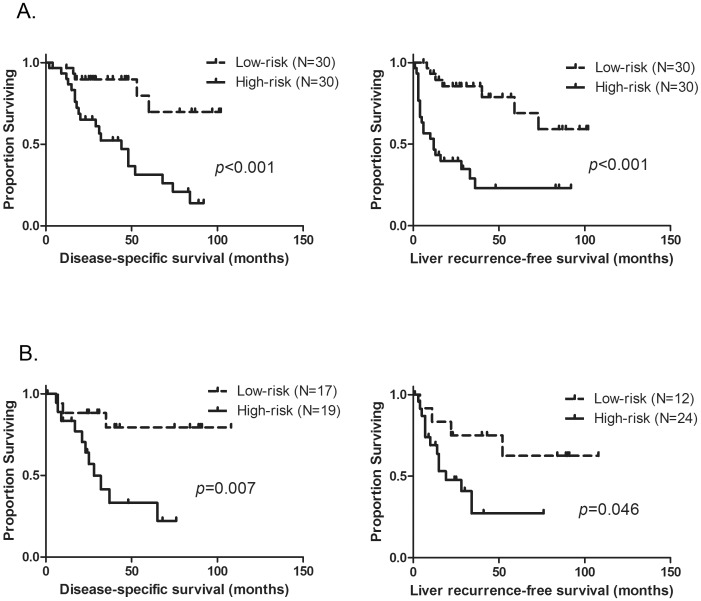
Disease-specific survival (DSS) and Liver recurrence-free survival (LRFS) of patients following curative liver resection stratified by molecular risk scores (MRS). A. Kaplan-Meier estimates of DSS (left panel) and LRFS (right panel) for the patients in high-risk and low-risk groups among the training set cohort (N = 60) B. Kaplan-Meier estimates of DSS (left panel) and LRFS (right panel) for the patients in high-risk and low-risk groups among the test set cohort; Of note, the threshold values to discriminate the high-risk and low-risk group were the same as used in the analysis for the training set cohort.

MRS’s were then calculated and patients were classified as high or low-risk for cancer death and liver recurrence, respectively. Among the 60 patients in the training set, patients in the high risk group had a significantly shorter DSS and LRFS compared with those in the low risk group (DSS: median 44 months vs not reached, and 3-year DSS rates, 52% vs 90%, *p*<0.001, LRFS: median 12 months vs not reached, and 3-year LRFS rates, 23% vs 86%, *p*<0.001, Figure 3A).

### Validation of MRS Using Independent Test Cohort

An independent test set of patients was used to validate the performance of the MRS as prognostic marker for cancer death and liver recurrence. The associations of clinicopathological variables with high- and low-risk signatures in both the training and test sets are summarized in table 2. There was no correlation between MRS, CRS, the use of chemotherapy before or after liver resection or regional chemotherapy using HAI, except for a statistically borderline correlation between MRS and CRS for LRFS (*p* = 0.054). Patients in the test set with a high MRS had shorter DSS and LRFS compared to those in the low risk group (DSS: median, 28 months vs not reached, and 3-year DSS rates, 42% vs 79%, *p* = 0.007, LRFS: median, 19 months vs not reached, and 3-year LDFS rates, 32% vs 80%, *p* = 0.046, respectively, Figure 3B).

**Table 2:** Association of clinicopathological variables with high- or low-risk signature for cancer death and liver recurrence in training and test cohort patient (N = 60/36).

Training set (N = 60)	Signature for cancer death					Signature for liver recurrence					
Variables	High risk (N = 30)		Low risk (N = 30)	*p*		High risk (N = 30)			Low risk (N = 30)		*p*
Tumor size >5 cm (%)	9 (30)		7 (23)	0.77		7 (23)			9 (30)		0.77
Primary N+ (%)	21 (70)		14 (47)	0.12		20 (67)			15 (50)		0.30
>1 liver tumor (%)	19 (63)		20 (67)	1.00		22 (73)			17 (57)		0.28
DFI <12 mo	21 (70)		11 (37)	0.02		21 (70)			11 (34)		0.02
CEA >200	4 (13)		1 (3)	0.35		4 (13)			1 (3)		0.35
CRS ≥3 (%)	15 (50)		8 (27)	0.11		15 (50)			8 (27)		0.11
Chemotherapy prior to surgery	20 (67)		23 (77)	0.57		23 (77)			20 (67)		0.57
Chemotherapy after surgery	22 (73)		26 (87)	0.33		25 (83)			23 (77)		0.75
HAI after surgery	10 (33)		9 (30)	1.00		9 (30)			10 (33)		1.00
**Test set (N = 36)**											
**Variables**	**High risk (N = 19)**		**Low risk (N = 17)**	***p***		**High risk (N = 26)**			**Low risk (N = 10)**		***p***
Tumor size >5 cm (%)	6 (32)		0 (0)	0.02		6 (23)			0 (0)		0.16
Primary N+ (%)	14 (74)		8 (47)	0.17		19 (73)			3 (30)		0.026
>1 liver tumor (%)	10 (53)		9 (53)	1.00		15 (58)			4 (40)		0.46
DFI <12 mo	10 (53)		9 (53)	1.00		15 (58)			4 (40)		0.46
CEA >200	1 (6)		2 (11)	1.00		3 (12)			0 (0)		0.55
CRS ≥3 (%)	9 (47)		5 (29)	0.32		13 (50)			1 (10)		0.054
Chemotherapy prior to surgery	15 (79)		11 (65)	0.46		20 (77)			6 (60)		0.41
Chemotherapy after surgery	16 (84)		15 (88)	1.00		23 (89)			8 (80)		0.60
HAI after surgery		5 (26)	10 (59)		0.09		9 (35)	6 (60)		0.26	

The concordance indices of the MRS as a continuous variable for DSS and LRFS in the analysis of the test set cohort were 0.71 and 0.70, while those of CRS for DSS and LRFS were 0.65 and 0.66. The MRSs performed better in discriminating patients at high and low risk of both DSS and LRFS compared with the CRS. Furthermore, in multivariate Cox-regression analysis including both MRS and CRS, MRS remained an independent and significant predictor of DSS and LRFS, indicating that the MRS provided additional prognostic information over the CRS. (Table 3).

**Table 3:** Univariate and multivariate analysis of DSS and LRFS.

	DSS			LRFS		
	UV *p*	MV *p*	HR (95% CI)	UV *p*	MV *p*	HR (95% CI)
CRS	0.018	0.048	3.05 (1.01–9.13)	0.010	0.037	3.18 (1.08–9.45)
MRS	0.00046	0.0012	13.9 (2.84–67.87)	0.008	0.027	8.68 (1.27–59.19)

UV, univariate, MV, multivariate.

### Combination of MRS and CRS Constitutes a better Prognostic Marker

**Figure 4:**
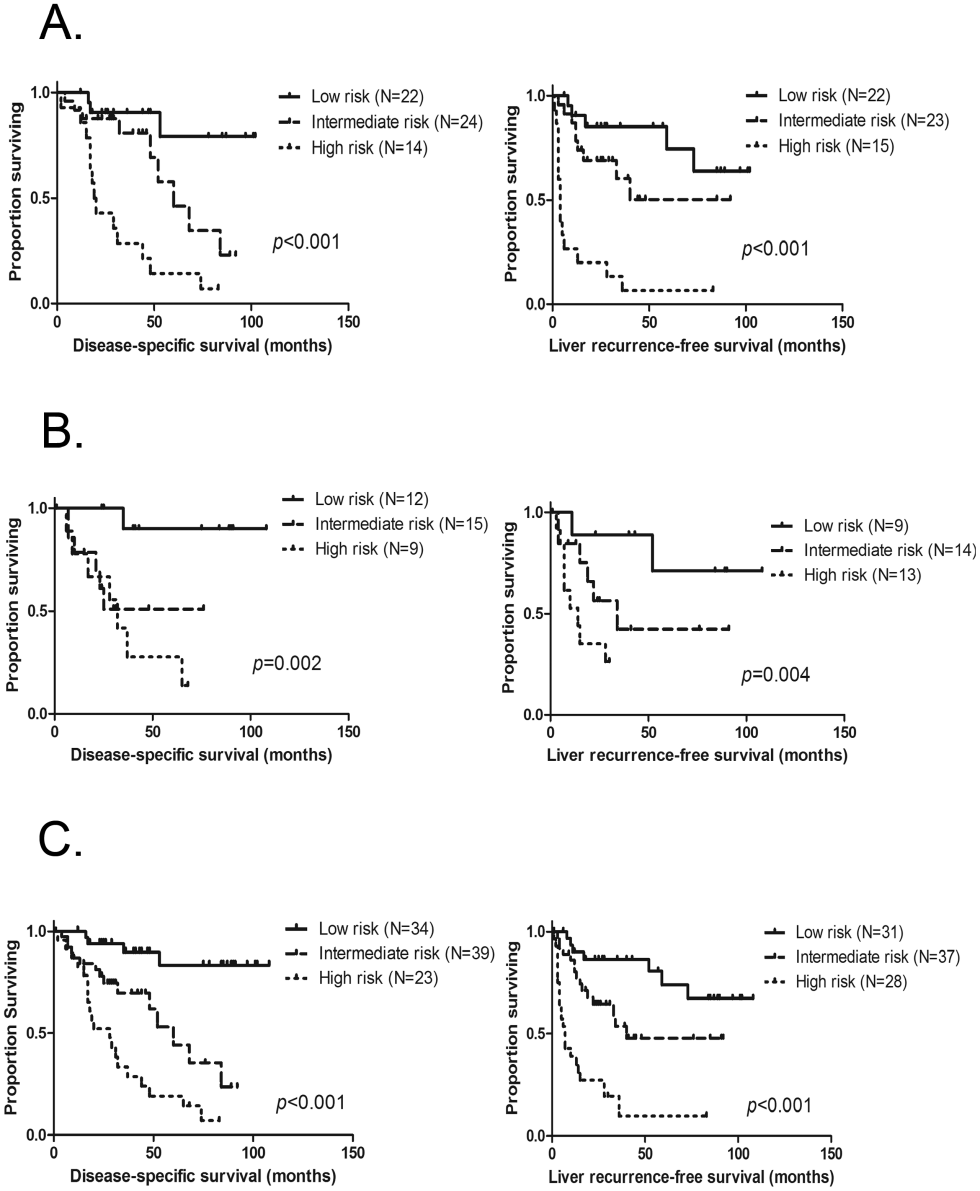
Risk stratification by combination of CRS and MRS for DSS and LRFS. A. Kaplan-Meier estimates of DSS (left panel) and LRFS (right panel) for patients in the high, intermediate, and low risk groups among the training set cohort (N = 60) B. Kaplan-Meier estimates of DSS (left panel) and LRFS (right panel) for patients in the high, intermediate and low risk group among the test set cohort (N = 36) C. Kaplan-Meier estimates of DSS (left panel) and LRFS (right panel) for patients in the high, intermediate and super-low risk group among the entire cohort (N = 96).

Since the MRS was shown to provide additional prognostic information for DSS and LRFS, independent of the CRS, we evaluated how well the combination of these two scores discriminated outcomes. We divided the patients, based on CRS and MRS, into three groups: high risk (CRS and MRS both high), low risk (CRS and MRS both low), and intermediate risk, (those who did not fit into these two groups). Based on this stratification, the survival curves for DSS and LRFS for these three groups were clearly separated in the analyses of both the training and test cohorts. For the training cohort patients (N = 60), the 3-year DSS/LRFS rates were 91/85% in the low risk group, while they were 81/60% and 29/13% in the intermediate and high risk group, respectively (*p*<0.001 for DSS and *p*<0.001 for LRFS). For the test cohort patients (N = 36), the 3-year DSS/LRFS rates were 90/89% in the low risk group, while they were 51/42% and 42/26% in the intermediate and high risk group, respectively (*p* = 0.002 for DDS and *p* = 0.004 for LRFS). When the same risk stratification scheme was applied to the entire cohort, patients in the low risk group had significantly longer DSS and LRFS compared to those in the intermediate risk and high risk groups: DSS; median, not reached vs 60 months vs 28 months and 3-year DSS rate, 90% vs 70% vs 34% (*p*<0.001), LRFS; median NR vs 40 months vs 7 months, and 3-year LRFS rates, 86% vs 54% vs 10% (*p*<0.001) for patients in high, intermediate and low risk groups, respectively (Figure 4).

## Discussion

Although uniformly classified as Stage IV disease by traditional TNM staging, hepatic MCRC is a heterogeneous disease with variable response to therapy and variable long-term outcomes. After complete resection, 10 year cure rates are approximately 20%, however, 20 to 30% of patients die of their disease within 2 years. [2], [27] Although multiple risk stratification schemes based on clinicopathological factors have been developed, none have been able to risk-stratify patients sufficiently enough to guide treatment decisions and most are not consistent when tested across different institutions. [3] Furthermore, prediction of recurrence patterns has been poor and this ability could have substantial implications for adjuvant therapy strategies. It is clear that we need better prognostic tools that can impact treatment decisions.

In the present study, we examined the global gene expression profiles of hepatic MCRC in patients who underwent a potentially curative liver resection and constructed MRSs to predict long-term outcomes after surgery. The MRSs were validated to be able to independently predict DSS and LRFS. Importantly, this prognostic ability was independent of our best clinical predictor, the CRS. When the MRS was used in combination with the CRS, we were able to identify subgroups of patients with extremely high and low risk for liver recurrence and death. Our findings indicate a great potential for gene signatures as a guide for personalized therapy for patients with potentially resectable liver MCRC.

One of the major challenges in applying clinical staging schemes for resectable hepatic MCRC is a great and growing clinical heterogeneity since new and more effective chemotherapy has been developed. Recent increased use of effective chemotherapy has expanded the indication for surgical therapy and made more patients potentially eligible for resection. As a result, the additional heterogeneity of treatment backgrounds has added to the complexity of this patient population further limiting the broad applicability and prognostic power of clinicopathological variables. Azye et al recently demonstrated a significant difference in the predictive power of several clinical risk scoring systems for patients with MCRC who did and did not receive chemotherapy prior to liver resection. Furthermore, in the patients who received chemotherapy prior to resection the clinical risk scoring systems were more accurate when measured after response to therapy was accounted for. [28] This observation further highlights the limitations of clinical staging schemes in the current era.

Despite the fact that 75% of our patients received various chemotherapy regimens as adjuvant or neoadjuvant therapy, the CRS was shown to be a reasonably accurate predictor of cancer death and liver recurrence (concordance indices 0.65 and 0.66, respectively). This finding is consistent with previously reported outcomes from our institution. [2], [6], [8] However, the CRS is imperfect for clinical use as a guide to individualize therapy for patients with resectable hepatic MCRC. First, the predictive ability of the CRS does not consistently predict outcomes in similar patient populations in different institutions. For example, Zakaria, et al reported a much lower concordance index (0.56) for our CRS when it was applied to their cohort of patients at the Mayo Clinic. [3] Secondly and more importantly, the difference in outcomes for patients with high and low CRSs is not great enough to impact clinical management. For example, the median survival of patients with a high CRS in this study was 38 months, making it difficult to recommend against resection for these patients. Therefore, our goal was to identify a genetic biomarker that could identify patients with such favorable or poor outcomes that clinical decisions could be rendered; avoiding futile surgery or performing surgery in patients with apparently poor clinical characteristics.

Global gene expression profiling in MCRC will likely provide greater insight into the underlying biology and clinical behavior of specific patients. It also seems likely that this genetic profiling will ultimately provide prognostic signatures that can dictate specific treatment regimens based on superior outcome prediction. It is not surprising that our MRS, based on gene profiling, was a powerful and independent prognostic tool for cancer-related death and recurrence patterns. A critically important finding in this study was that there was a significant discrepancy in risk-stratification by CRS and MRS and that these prognostic scores were independently predictive based on a multivariate analysis. For example, among the patients who classified as high risk for cancer death by MRS, more than half (53%) of them was classified as low risk by CRS (<3). This finding indicates that combining clinical and molecular prognostic tools may be the most powerful method of predicting outcome and impacting treatments decisions for patients with resectable hepatic MCRC.

In addition to cancer-specific death, overall and liver recurrence was chosen as one of the end points of treatment failure in the current study. No accurate predictor of overall RFS could be determined. Although the reason why a gene signature failed to be associated with overall recurrence is not clear, one possible explanation is inaccuracy in diagnosis of extrahepatic recurrence, especially in lymph nodes. Tsunoda et al. reported only 75% of accuracy in diagnosis of lymph node metastasis of colorectal cancer by PET/CT. [29] It should be noted that in this study, the recurrence was defined based on imaging studies and histological confirmation was not necessary obtained. Although HAI chemotherapy is well known to reduce liver recurrence after hepatectomy for MCRC [19], [30], it has not been universally accepted due to the difficulty in administering this therapy and concerns over biliary toxicity. [31] Accurate prediction of liver-specific recurrence would be useful to select patients most likely to benefit from adjuvant regional therapies such as HAI chemotherapy. In this study, one third of our patients received HAI chemotherapy as adjuvant therapy. However, the fraction of patients who received HAI chemotherapy was not higher in the group with a high MRS for liver recurrence than in the one with a low MRS. As was the case for DSS, the CRS and MRS were independent of each other in their ability to predict liver recurrence and were powerful in combination in predicting this event.

MRS provides additional prognostic information over the CRS and the combination of these two significantly improves risk stratification following liver resection. Among the entire cohort, 90% of the patients classified as high risk by both CRS and MRS developed liver recurrence and more than 65% died within 3 years, while more than 85% of those classified low risk both by CRS and MRS were liver recurrence free and 90% were alive at 3 years. Identification and further validation of such high and low risk groups could lead to consideration of individualized therapy for these patients. For example, non-operative approaches could be considered for patients at high risk of early death and adjuvant regional strategies such as HAI chemotherapy could be considered for patients at high risk of liver recurrence. Conversely, patients with a low risk of failure could be considered for surgery alone without additional chemotherapy. Prospective evaluation of the use of such powerful prognostic data on the selection of patients for novel therapeutic approaches is warranted.

The genes that constitute the risk scores were selected in a purely statistical manner from the pool of genes in the array, and a mechanistic role in colorectal cancer progression has not been defined for many. However, some of the selected genes have been shown to play an important role in cancer biology. For example, *FGFBP1* (fibroblast growth factor binding protein 1) was identified as one of the genes with the highest impact on both DSS and LRFS. This gene is shown to play a critical role in promoting tumor angiogenesis in various cancers including colorectal. [32] Another predictive gene identified in this study was *BAG3* (BCL-2 associated athanogene 3) which is known to have anti-apoptotic effects in cancer cells and enhance resistance to chemotherapy. [33] The list of predictive genes we have identified may also provide insight and direction into the study of the biology of resectable metastatic colorectal cancer to the liver, as well as provide potential new targets for therapeutic development. On the other hand, it is important to note that this study was not designed to explore related biological pathways and that the expression signatures do not include all relevant genetic events to explain underlying tumor biology.

Limitations of the current study include its retrospective nature, selection bias of the tumor samples and limited sample size. Due to the technical limitations of the Illumina gene chip, only patients with banked frozen tissue could be included. Additionally, some of the tissue samples with poor quality RNA were excluded in order to obtain accurate data. In fact, 31% of our patients were excluded from this study because the tumor did not contain enough viable cells on screening histological examination. Furthermore, tumors with a radiologic complete response to chemotherapy were not in our tumor bank. Therefore, prospective evaluation of the MRS using tissues obtained prior to neoadjuvant chemotherapy is an important consideration. Fortunately, newer technology that allows genomic profiling of partially degraded samples, such as formalin-fixed tissue [18], [34] is being developed and used. Furthermore, the performance of current hybridization-based array technologies continues to improve, while costs decrease [33], [34]. The genetic heterogeneity of multiple metastatic tumors may be another limitation of this study. The majority of the patients had multiple tumors but only one tumor per patient was assayed. Two studies evaluating the gene expression profiles both of primary colorectal cancer and corresponding hepatic metastasis have demonstrated remarkably similar gene expression profiles between the primary and metastatic tumors. [35], [36] Given the similarity in gene profile between primary and metastatatic tumors it is likely that the gene expression of multiple liver metastases in a single patient are similar. Chemotherapy prior to liver resection theoretically might alter the gene expression of metastatic tumors. However, this has never been definitively proven and does not compromise the prognostic importance of MRS, as we directly correlated the snap shot of gene expression profile at the time of surgery (regardless of prior chemotherapy) with long-term outcomes. Furthermore, the MRSs among patients who received chemotherapy prior to liver surgery were not significantly different from the MRSs of those who did not.

With further improvements in technology and the clear need for better prognostication in cancer, molecular predictive tools will very likely play a more prominent role. Further clinical validation of the MRSs developed in our study will be needed before it is introduced into clinical practice and such technological advances will make large-scale validation studies possible. A custom gene chip of the 19 genes associated with survival in the current study may be useful in the complete staging of patients with resectable MCRC isolated in liver. Molecular staging incorporating MRSs with other clinicopathological features will likely provide better and clinically meaningful risk-stratification for patients with hepatic MCRC than current TNM staging.

In summary, this global gene expression analysis of tumors from patients undergoing hepatectomy for MCRC allowed us to construct a novel molecular marker to predict long-term prognosis. These molecular risk scores predicted liver recurrence and cancer death better than and independent of the clinical risk score. Furthermore, the combination of the CRS and MRS provided very powerful and clinically meaningful prediction of outcomes. While further validations of these gene signatures using larger multi-institutional cohorts are needed, these new staging systems incorporating clinicopathological and genetic variables may provide more accurate risk stratification, and the potential for individualized therapy.

## Supporting Information

Table S1 **Lists of genes selected to construct MRS for DSS and LRFS.**
(XLSX)
